# Toxicity of Essential Oils of *Origanum vulgare*, *Salvia rosmarinus*, and *Salvia officinalis* Against *Aculops lycopersici*

**DOI:** 10.3390/plants14101462

**Published:** 2025-05-14

**Authors:** Thomas Giordano, Giuliano Cerasa, Ilaria Marotta, Mauro Conte, Santo Orlando, Adele Salamone, Michele Massimo Mammano, Carlo Greco, Haralabos Tsolakis

**Affiliations:** 1 Department of Agricultural, Food and Forest Sciences, University of Palermo, Viale delle Scienze, Bldg. 4, 5, 90128 Palermo, Italy; thomas.giordano@unipa.it (T.G.); giuliano.cerasa@unipa.it (G.C.); ilaria.marotta02@unipa.it (I.M.); mauro.conte@unipa.it (M.C.); santo.orlando@unipa.it (S.O.); 2 CREA, Research Centre for Plant Protection and Certification, 90133 Palermo, Italy; adele.salamone@crea.gov.it (A.S.); massimo.mammano@crea.gov.it (M.M.M.)

**Keywords:** medicinal and aromatic plants (MAPs), precision agriculture, essential oils, tomato russet mite

## Abstract

The tomato russet mite (TRM), *Aculops lycopersici*, is a destructive pest of tomato crops worldwide. It poses a significant challenge to growers in both greenhouse and open-field conditions. Traditional chemical control methods are often ineffective, promote resistance, and have negative environmental impacts. This has prompted the search for alternative strategies, such as biological control and eco-friendly botanical pesticides. In this study, we evaluated the acaricidal effects of essential oils (EOs) extracted from three officinal plants, *Origanum vulgare* L., *Salvia rosmarinus* Spenn., and *Salvia officinalis* L., cultivated using precision aromatic crop (PAC) techniques. Their efficacy was evaluated against *A. lycopersici* under laboratory conditions. The chemical composition of the EOs was determined by solid-phase microextraction (SPME) coupled with gas chromatography–mass spectrometry (GC-MS). The dominant component of *O. vulgare* EO was carvacrol (83.42%), followed by ρ-cymene (3.06%), and γ-terpinene (2.93%). In *S. rosmarinus*, α-pinene (28.0%), 1,8-cineole (11.00%), and borneol (7.72%) were the major components. *S. officinalis* EO was characterized by high levels of 1,8-cineole (27.67%), camphor (21.91%), and crisantenone (12.87%). We tested multiple concentrations (320–5000 μL L−^1^) and exposure times (1–4 days) to assess mite mortality. The results revealed both dose- and time-dependent toxic activity, with significant differences among EOs. *O. vulgare* EO was the most toxic, causing 90% mortality at 0.5% (*w*/*v*) concentration after 4 days. *S. rosmatinus* and *S. officinalis* EOs had more limited effects, with 46% and 42% mortality, respectively. Lethal concentration (LC_50_) values were 2.23 mL L−^1^ (95% CI: 1.74–3.05) for *O. vulgare*, 5.84 mL L−^1^ (95% CI: 3.28–22.29) for *S. rosmarinus*, and 6.01 mL L−^1^ (95% CI: 2.63–261.60) for *S. officinalis*. These results indicate that *O. vulgare* EO shows efficacy comparable to commercially available botanical pesticides. Our findings support the potential of *O. vulgare* EO as a viable alternative for the control of *A. lycopersici*, contributing to integrated pest management (IPM) strategies.

## 1. Introduction

The tomato russet mite (TRM), *Aculops lycopersici* (Tryon, 1917) (Acari: Eriophyidae), is a worldwide distributed pest, present in both tropical and temperate regions [1,2]. It infests mainly solanaceous plants, including wild species like black nightshade *Solanum nigrum* L. as well as cultivated crops such as pepper (*Capsicum annuum* L.), eggplant (*Solanum melongena* L.), potato (*Solanum tuberosum* L.), and tomato (*Lycopersicon esculentum* Miller). *Aculops lycopersici* is especially damaging to tomato cultivation, affecting yields in greenhouses and open-field crops. The eriophyid feeds on leaf, stem, and fruit surfaces, resulting in significant cellular disruption. Mite feeding damages the adaxial and abaxial epidermal cells of leaves, causing the development of a dense layer of callous tissue near the parenchyma where cell death occurs [3]. The feeding behavior of *A. lycopersici* causes russeted leaves, stems, and fruits and, in severe cases, can lead to plant death [4]. Recent studies suggest that *A. lycopersici*, similar to other eriophyid mites, may also transmit virus vector, increasing its overall negative impact on crops [5].

Thriving in warm, arid conditions, *A. lycopersici* populations can escalate rapidly, causing substantial damage. These mites typically cluster and feed where they initially land on the plant, only spreading upward once the population density increases. Infestations often remain undetected until visible symptoms emerge; at this point, a large population has already built up, especially on drought-stressed plants that accelerate mite reproduction [6], as tomato plants under drought stress accumulate proteins and amino acids beneficial to the mite, while their jasmonic acid-mediated defense responses are weakened [7]. Recently, infestations by *A. lycopersici* have surged in European tomato crops, echoing a broader trend of increasing economic impact from eriophyoid mites worldwide [8].

Synthetic acaricides have long been the standard approach for controlling phytophagous mites; however, the development of resistance, along with harmful effects on native phytoseiid populations, has raised significant concerns [9]. These challenges have prompted increasing interest in biological alternatives for integrated pest management (IPM), as researchers seek more sustainable and environmentally friendly solutions. The use of plant-based biopesticides, including essential oils, is increasingly promoted within the EU IPM framework as a viable tool to reduce dependency on synthetic pesticides [10]. Efforts to identify natural enemies for controlling tomato russet mites have led to a substantial list of potential predators, particularly within the Phytoseiidae family [11,12,13,14,15]. Several species of these predatory mites have been observed in natural association with tomato russet mites, able to feed and reproduce on them, at least under laboratory conditions [16,17]. However, when tested under field conditions, predators often failed to suppress the eriophyid population or required several generations for adapting to tomato plants, limiting their impact towards the pest population [12,13,18,19,20]. Moreover, the effectiveness of these predators can be further hindered by trichome density and pollen availability [21]. Tomato trichomes hamper predator movement and provide shelter for russet mites, while toxic secondary metabolites of plants and prey can intoxicate the predators, making biological control even more complicated [8,21].

Botanical pesticides have gained renewed attention as a cost-effective alternative to synthetic chemicals in integrated pest management (IPM) [22]. They were commonly used prior to World War II, but their use declined with the rise of synthetic pesticides after the war [22,23,24,25]. In contrast, botanical products usually have fewer or less severe negative effects on human health and the environment, support natural enemies of pests, and exhibit low environmental persistence [10]. Among the botanical solutions, essential oils (EOs) and their active compounds have emerged as promising biocides, particularly for their efficacy against arthropod pests, including tephritid flies, ambrosia beetles, mites, ticks, and even certain weeds [26,27,28,29]. The large-scale production of plant-derived EOs for the perfume and flavoring industries also makes these substances commercially viable [29]. Current research on botanical pesticides frequently investigates plant families such as Apiaceae, Myrtaceae, Lamiaceae, Meliaceae, Annonaceae, Simaroubaceae, and, more recently, Asteraceae [25,30]. In particular, extracts derived from the Lamiaceae family have demonstrated significant acaricidal activity against mites such as *Tetranychus urticae* Koch, as well as insecticidal effects on pests including *Bemisia tabaci* (Gennadius) and *Lasioderma serricorne* (Fabricius) [31,32,33,34], thereby reinforcing the relevance of this plant family in pest management strategies.

The present study aimed to assess the acaricidal effects of essential oils from *Origanum vulgare* L. (oregano), *Salvia rosmarinus* Spenn., (=syn. *Rosmarinus officinalis* L.) (rosemary), and *Salvia officinalis* L. (sage) (Lamiaceae) cultivated using PAC techniques, on *A. lycopersici* under laboratory conditions.

## 2. Results

### 2.1. Precision Aromatic Crop (PAC) Techniques

**Figure 1:**
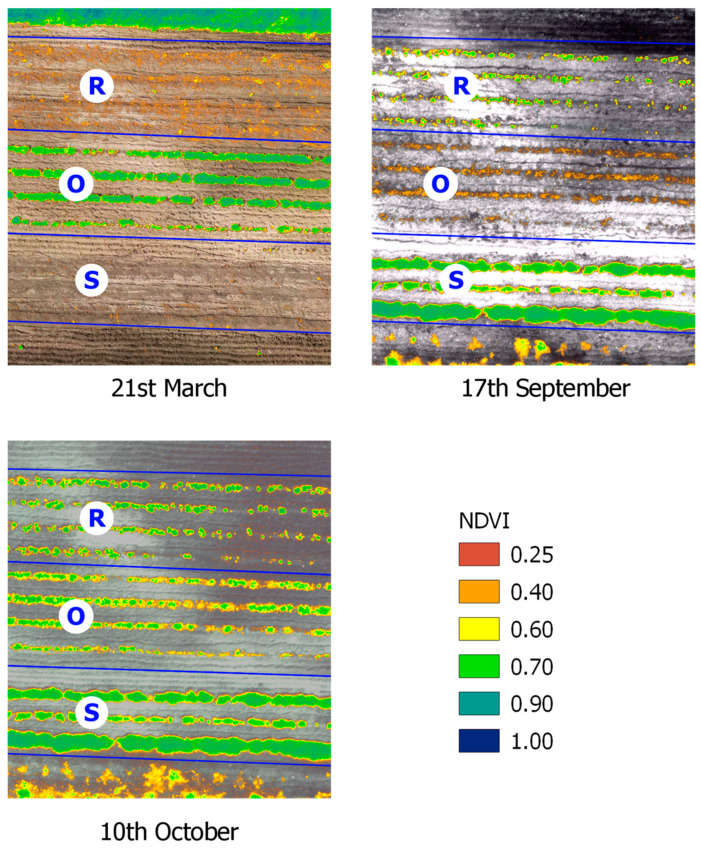
MAPs NDVI values to determine harvesting time. (R = Rosemary; O = Oregano; S = Sage).

**Figure 2:**
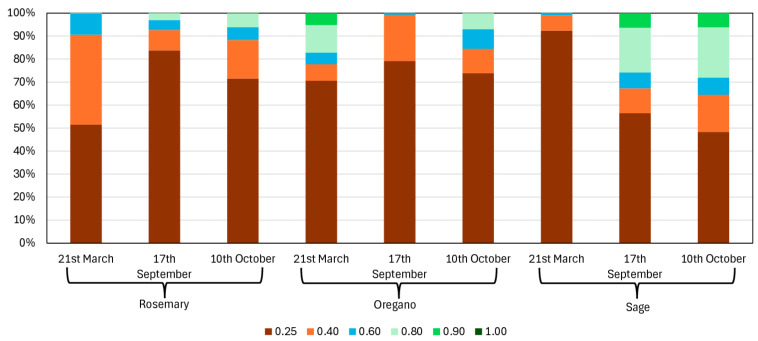
Percentage surface area of rosemary, oregano, and sage based on NDVI classes during the three considered periods.

In, oregano was in its growth phase, while rosemary remained bare and sage exhibited low vigor. In September, 2024, oregano had been harvested, leaving bare soil with visible clumps, whereas rosemary displayed medium vegetative vigor and sage reached full vigor. In October, 2024, sage maintained full vigor, and rosemary continued developing, with vigor classes increasing to 0.60–0.80 (oregano had resumed growth following the rains). More than 50% of the sage surface showed NDVI values greater than 0.40 (Figure 1 and Figure 2).

Multispectral analysis enabled the identification of the optimal harvest period, corresponding to mean NDVI values of 0.85 for rosemary (in May), 0.80 for oregano (in June), and 0.75 for sage (in May and October 2024).

### 2.2. Analyses of Essential Oils

The essential oils extracted from the three species had a light-yellow color and a characteristic aroma. The volatile profile of oregano EO includes 27 volatile compounds belonging to the following phytochemical groups: monoterpene phenol (84.31%), monoterpene hydrocarbons (9.53%), oxygenated monoterpenes (1.91%), sesquiterpene hydrocarbons (2.87%), ethers, ketones, and alcohols each contributing less than 1%. The principal component of this EO was carvacrol, followed by ρ-Cymene and γ-Terpinene. Nineteen main components representing 89.3% of the rosemary essential oil were detected: 47.53% of monoterpene hydrocarbons, 33.37% of oxygenated monoterpenes, and 8.27% of sesquiterpene hydrocarbons (Table 1). As shown in Table 1, the sage EO yield was 0.29%; a total of 23 volatile compounds were identified by GC-MS. These compounds were categorized into monoterpene hydrocarbons (29.64%), oxygenated sesquiterpenes (2.81%), oxygenated monoterpenes (59.57%), and sesquiterpene hydrocarbons (2.72%). Additionally, other compounds not classified in the previous categories were also identified. Monoterpene hydrocarbons and oxygenated monoterpenes were the most abundant compounds in all sage leaf samples, accounting for more than 90% of the total identified compounds. Among the monoterpene hydrocarbons, camphene and crisantenone were the most prevalent (average of 9.26% and 12.87%, respectively), while eucalyptol and camphor were the dominant compounds among the oxygenated monoterpenes (average of 27.67% and 21.91%, respectively) [35].

### 2.3. Toxicity of O. vulgare, S. rosmarinus, and S. officinalis EOs Against A. lycopersici

The three factors compared in the GLM analysis were *EO* (oregano, rosemary, sage), *Concentration* (0, 320, 640, 1280, 2500, 5000) and *Time* (1, 2, 3, 4 days). The three EOs tested showed significantly different effects on *A. lycopersici* adults (F*_2, 648_* = 5.75, *p* = 0.003), and a significant different effect was registered both for the factors *Concentration* (F*_5, 648_* = 124.45, *p* < 0.001) and *Time* (F*_3, 648_* = 61.67, *p* < 0.001). The interaction between the first two factors (F*_6, 648_* = 18.42, *p* < 0.001) and between *Concentration* and *Time* (F*_15, 648_* = 2.14, *p* = 0.007) indicate that each EO concentration caused different toxic effects, and that the mortality had a different trend in the three adopted EOs during the test period.

The highest concentration of oregano (*O. vulgare*) EO (5000 μL L−^1^) hardly affected *A. lycopersici* after 24 h (66% of mortality), and high mortality (90%) after 4 days was registered, while it was significantly lower with concentration of 2500 μL L−^1^ and almost null in all the remaining concentrations (Table 2). For rosemary (*S. rosmarinus*) and sage (*S. officinalis*), the acaricidal effect remained scarce, with marginal increases during time even at the highest concentrations. No statistical differences were noted between each considered concentration for the latter extracts. As a matter of fact, only the highest concentration of oregano EO can be classified as highly toxic (class 4) according to the toxicity categories proposed by Hardman et al. [36]. At the concentration of 2500 μL L−^1^, the effect was moderate (class 2), while at lower concentrations (1280, 640, 320 μL L−^1^), the efficacy was classified as non-toxic (Class 1). Rosemary and sage EOs caused similar results, falling in classes 1 and 2 (Table 2).

The analysis of mean survival times further corroborated these trends, indicating that at the highest concentration of 5000 μL L−^1^ of oregano EO, the mean survival time of *A. lycopersici* adults was about one day after treatment. A higher survival time was recorded using 2500 μL L−^1^ of the latter EO, although it was statistically lower compared to the control (Table 2). In contrast, survival times at lower concentrations did not differ to those observed in the control tests. This highlights that the acaricidal efficacy of oregano EO is evident only surpassing the critical concentration threshold of 2500 μL L−^1^. In fact, the chemotype classification of essential oils plays a key role in EO selection, as different chemotypes may contain varying concentrations of active compounds that can influence acaricidal efficacy. By understanding the chemotype, we can optimize the concentration needed to achieve the desired effects, ensuring both effective pest control and minimal environmental impact.

The lethal concentration for each of the three essential oils was calculated by probit analysis. The Pearson goodness-of-fit test indicated that LC values obtained by oregano EO do not fit the linear model (*χ^2^* = 21.62; *p* < 0.001), because of the high discrepancies of toxic effects registered at the highest dose (90% of mortality) and the lower doses (less than 36% of mortality). On the other hand, LC values calculated for the other two EOs were well fitted to the linear model (Table 3). 

**Figure 3:**
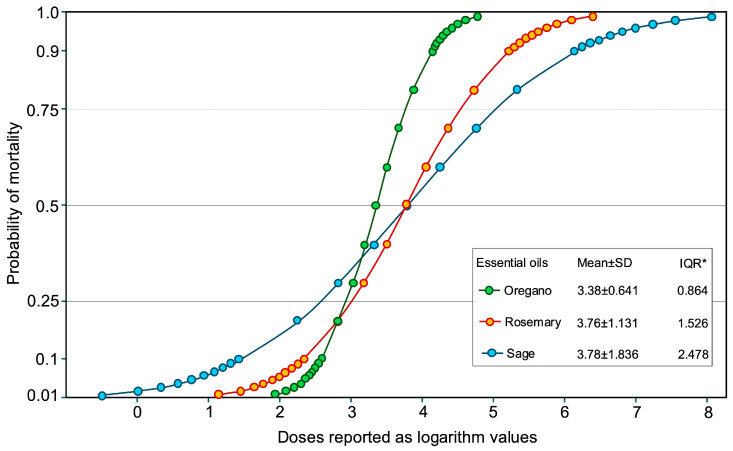
Parametric cumulative failure plot for probability of mortality based on Probit data and maximum likelihood method. * Interquartile range.

The LC_50_ values were 2.23 mL L−^1^ for oregano EO, 5.84 mL L−^1^ for rosemary EO, and 6.01 mL L−^1^ for sage EO. The probability plot built on probit data indicates a more sensitive response for mortality in oregano EO doses, in comparison to the other two EOs (IQR = 0.864). The probability of 50% of mortality was almost the same for rosemary and sage EOs but the different values of IQR (1.526 and 2.478 for the latter EOs, respectively) indicate a more immediate effect of rosemary EO than of the sage EO (Figure 3).

## 3. Discussion

In this study, the correlation between NDVI values and harvest timing for medicinal and aromatic plants such as oregano, sage, and rosemary was validated focusing on the establishment of NDVI thresholds for optimal plant stages. NDVI, a non-destructive index measuring plant canopy reflectance in the red and near-infrared spectra, is widely used to assess plant health and vigor, correlating with biomass and nutritional content [37]. Previous studies have demonstrated NDVI’s effectiveness in predicting optimal harvest times in various crops; for instance, NDVI values at specific phenological stages have accurately estimated yields in white oats [38,39]. Similar research on oregano indicates that phenological stages, such as full bloom, significantly influence herbage yield and essential oil content [40,41]. The determination of NDVI thresholds typically involves field experiments and regression analysis, correlating NDVI with plant health indicators. In cotton, for example, NDVI thresholds were linked to nitrogen fertilizer levels and yield [42]. Though hyper-tuning of NDVI thresholds for medicinal and aromatic plants is not explicitly discussed in the reviewed studies, iterative testing and optimization techniques, as demonstrated in vineyards [43], are likely applicable.

Numerous studies support the use of NDVI thresholds as reliable indicators for determining optimal harvest periods. Analysis of NDVI time series reveals that sharp and sustained decreases in NDVI values effectively signal harvest readiness, as demonstrated in sugarcane [44]. In rice, NDVI values at specific phenological stages, particularly the minimum slope during maturation, can predict harvest timing accurately [45]. In vineyards, NDVI patterns observed 15 to 20 days prior to veraison show strong correlation with actual harvest dates, enabling early prediction of optimal harvest windows [46]. Additionally, in crops like winter wheat and spring barley, peak NDVI values during the heading phase, modeled through polynomial regression, have been used successfully to forecast yield and harvest periods [47]. The dynamic threshold method further refines harvest predictions by adjusting NDVI thresholds to account for field management activities and crop phenology, enhancing accuracy [48]. Remote sensing approaches utilizing NDVI curves have also proven effective in monitoring crop development and accurately predicting harvest times, exemplified in summer maize [49]. Collectively, these findings underscore that NDVI thresholds, whether static or dynamic, serve as robust tools for identifying optimal harvest periods across diverse cropping systems.

For sage, the highest fresh herbage yield is achieved at the mature seed stage during spring harvest, with the optimal timing for a subsequent harvest occurring 70 to 100 days later [50]. Although NDVI is not explicitly mentioned, its monitoring could facilitate the identification of these developmental stages by reflecting changes in plant vigor. In rosemary, harvest periodicity and nitrogen concentration significantly influence leaf growth and essential oil yield, with higher yields obtained through fewer harvests at specific nitrogen levels [51]. NDVI could serve as a valuable tool to monitor growth dynamics and optimize harvest timing. Similarly, in oregano, season extension techniques such as low tunnels within high tunnels (LtHts) have been shown to enhance herbage and essential oil production, even under challenging climatic conditions [52]. The application of NDVI could aid in assessing the effectiveness of these methods and determining the most suitable harvest periods.

The three Lamiaceae species used in the present study are widespread in the Mediterranean basin and used from millennia for their therapeutic and culinary properties [53,54]. Moreover, the three species are included in the BELFRIT list [55], making easier a potential future biopesticide registration.

Oregano EO is mainly composed by carvacrol, thymol, and monoterpenes [56]. However, the predominant compound identified in this study was carvacrol, accounting for about 83% of the total composition. This confirms monoterpenoids as the dominant class of volatile organic compounds (VOCs) in oregano EO. This composition places this EO within the carvacrol oregano chemotype, primarily due to its high carvacrol content (~80%). Similar volatile profiles have been reported for oregano from other Mediterranean regions, suggesting that the essential oil composition is more strongly influenced by the oregano variety than by the geographical origin of cultivation [57].

Recent research has shown that carvacrol, along with other monoterpenoids, is highly toxic to a range of invertebrate pests, including insects, acari, and nematodes [58,59,60]. Furthermore, these compounds are environmentally friendly, as they biodegrade or dissipate quickly, and exhibit low toxicity to mammals, fish, and other non-target organisms [61]. These properties make monoterpenoids like carvacrol a viable alternative to synthetic pesticides for pest management.

The percentage composition of key components in rosemary EO indicates that α-pinene, 1,8-cineole, and borneol are the major constituents, as previously reported [62]. Furthermore, other researchers have noted that each rosemary EO typically contains nine major compounds, collectively accounting for over 90% of its composition, with 1,8-cineole being the predominant component (>52%) [63].

From a chemical perspective, rosemary EO can be classified into distinct chemotypes based on the relative abundance of its primary constituents. The main chemotypes identified include *cineoliferum*, characterized by a high 1,8-cineole content, *camphoriferum*, with camphor levels exceeding 20%, and *verbenoniferum*, where verbenone exceeds 15%. Additionally, chemotypes with high levels of α-pinene have been identified in specific regions, including Italy and Morocco [64].

The biological activity of α-pinene has been extensively investigated. In addition to its well-documented antifungal, antibacterial, and antiviral properties, α-pinene exhibits insecticidal and nematocidal effects [65], underscoring its potential for various control activities. According to a review by Jankowska et al. [66], α-pinene was found to be one of the most effective volatile organic compounds (VOCs) in inhibiting acetylcholinesterase (AChE), which may explain the higher entomotoxic activity of rosemary EO, in which α-pinene is generally the second most abundant compound. Moreover, several other constituents of rosemary EO, such as camphor, eucalyptol, and α-pinene, have been reported to exert a cytotoxic mode of action, leading to cell membrane damage [67,68].

The *Salvia* genus comprises about 900 species mainly distributed throughout the world, and some of which are used also in perfumery and cosmetics. The essential oil of many species of sage is characterized by the presence of 1,8 cineole (eucalyptol), β-thujone, camphor, borneol, and ρ-cymene which is attributed with the antimicrobial activity against many microorganisms [69,70].

The insecticidal efficacy of sage EO is attributed to its richness in monoterpenoids, which are widely recognized for their potent insecticidal effects against a broad spectrum of insects. For example, 1,8-cineole and α-pinene, two key monoterpenoid constituents of this EO, have been shown to inhibit erythrocyte acetylcholinesterase activity [71]. Moreover, it has been demonstrated that sage EO exhibits significant insecticidal activity on *Spodoptera littoralis* Boisduval, with mortality increasing proportionally to the concentration after 24 h of exposure [72]. It has also been demonstrated that essential oils of sage EO possess both contact toxicity and repellent effects on *Tetranychus urticae*. Generally, the terpenoids in essential oils exert various effects on insects, including toxicity, reduced maturity, and diminished reproductive capacity. These oils are also a complex mixture of neurotoxic compounds with selective action on insects, acting by interfering with the octopaminergic transmitters in arthropods. The action of these essential oils is likely due to the synergy or antagonism between the major compounds [73].

Interest in the use of plant extracts and essential oils as tools for controlling phytophagous populations has recently increased considerably, due to their ability to reduce environmental impact and preserve non-target organisms [10,29,74]. However, research has mainly focused on a limited number of phytophagous species, neglecting others that, although less studied, may be just as harmful or even more damaging. Regarding Acari, studies on vegetal products have mainly focused on *T. urticae* [25,31,75,76], while the knowledge available on the effectiveness of biopesticides against other damaging pests such as the tomato rust mite is still very limited. To the best of our knowledge, this is the first study on the effects of essential oils against *A. lycopersici*, as no previous research on this topic was found in the available literature.

Among the tested oils, oregano EO exhibited the highest acaricidal activity. However, significant efficacy was observed only at the highest concentration (5000 µL/L), suggesting that its toxic effect manifests only beyond a specific concentration threshold. Furthermore, the probit regression does not fit the proposed linear model, suggesting that the calculated LC50 does not represent the appropriate value. Probably, the main toxic effect of oregano EO is attributed to carvacrol, as it exhibits various bioactive properties, including antioxidant effects, inhibition of antibiotic-resistant bacteria, suppression of microbial and fungal toxins, and potential anticancer activity [57]. However, the primary mode of action of carvacrol remains unclear, although it has shown limited acetylcholinesterase inhibition in certain insect species, such as house flies, ticks, and cockroaches [71].

Regarding rosemary and sage EOs, Laborda et al. [31] assessed high toxic effects after 24 h exposure (79 to 100% of mortality) against *T. urticae* at concentrations ranging from 1500 to 2500 µL/L. Our results on *A. lycopersici* revealed very low toxicity after 24 h (2 to 12%) and also after 4-days exposure (26 to 54% of mortality), at comparable concentrations (1280 and 2500 µL/L). However, the sage EO used by the abovementioned authors had a different chemical profile: the main component was α-Thujone (42.3%), followed by camphor (11.0%) and 1,8-Cineole (10.3%); the latter two were the principal components of our sage EO (21.91 and 27.67% for the two components, respectively), while camphor was not detected in our sage EO. The rosemary EO used by Laborda et al. [31] also showed a different compound composition to that used in our experiments. The four main components of their rosemary EO were 1,8-cineole, α-pinene, camphor, and camphene (26.7, 18.6, 17.5, and 11.8 for the four compounds, respectively). However, concentration of the above compounds in our rosemary EO was quite different (11.0, 28.0, 6.2, and 7.0 for the above components, respectively). The lower toxic effects on *A. lycopersici* could be attributed to the different concentrations of the components of the essential oils adopted but also to a different susceptibility of the two mite species. Although essential oils from *O. vulgare*, *S. rosmarinus*, and *S. officinalis* show promising acaricidal properties, they could be particularly useful in early infestations to prevent population growth. However, there are currently no commercial products specifically available for the control of *A. lycopersici*. Further research on formulation and field efficacy is needed to assess their potential use in integrated pest management strategies, where natural alternatives can offer a sustainable option compared to chemical pesticides.

### Challenges in Field Application

Although essential oils demonstrated good efficacies in controlling the tomato russet mite in laboratory conditions, there are significant challenges in their application in field conditions. The main obstacles in the use of essential oils in field applications are related to their high photolability and thermolability, which limit their stability and efficacy over time [77]. Therefore, it is necessary to investigate the use of carrier systems capable of encapsulating the active ingredients and ensuring their controlled and prolonged release.

## 4. Materials and Methods

### 4.1. Cultivation of Officinal Plants with Precision Aromatic Crops (PAC) Techniques, and Essential Oil Extraction Methods

#### 4.1.1. Plants Cultivation Method

Cultivation of *O. vulgare* (Oregano), *S. rosmarinus* (Rosemary), and *S. officinalis* (Sage) was carried out at Morreale’s Farm in Grotte (Agrigento Province, Italy) (37°22′52.284″ N 13°40′24.067″ E) (World Geodetic Coordinate System 1984). The soil moisture regime is xeric, bordering on aridic, and the temperature regime is thermic. The experimental field includes 1520 plants of *O. vulgare*, 1980 plants of *S. rosmarinus*, and 2485 plants of *S. officinalis*, arranged with 35 cm spacing along the rows and 180 cm between the rows. Periodically, inter-row surface tillage was performed to control weed growth and disrupt soil capillarity. No organic or mineral fertilization was carried out during the growth period. The three species were cultivated under dry conditions. The seedlings were transplanted, and manual harvesting was performed for sage and rosemary, while for oregano, a combine harvester was used [78]. All species were cultivated simultaneously under identical environmental conditions.

#### 4.1.2. Precision Aromatic Crop (PAC) Techniques

**Figure 4:**
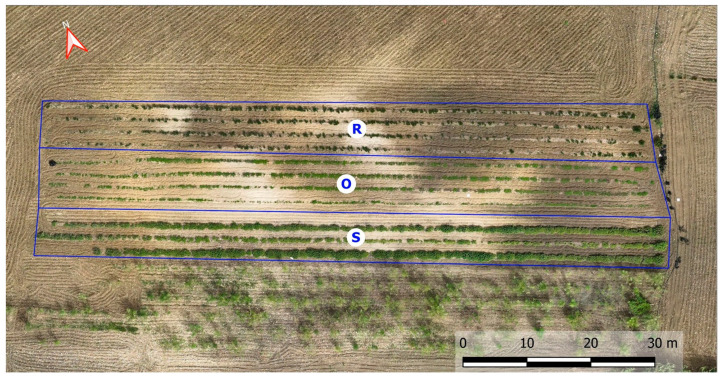
MAPs field monitoring through precision agriculture technologies (R = Rosemary; O = Oregano; S = Sage).

Precision Aromatic Crop (PAC) techniques were applied to optimize the cultivation and monitoring of medicinal and aromatic plants (MAPs) (Figure 4). Specifically, unmanned aerial vehicles (UAVs) equipped with multispectral cameras, combined with post-processing software, have become a widely adopted technique for assessing vegetation indices (VIs) in the management of MAPs. This method seeks to modernize agricultural practices by minimizing resource use and boosting productivity [79,80,81].

Advanced technologies for spatially variable crop condition monitoring, focusing on the use of UAVs equipped with multispectral cameras and a spectroradiometer to assess MAPs, were adopted.

**Figure 5:**
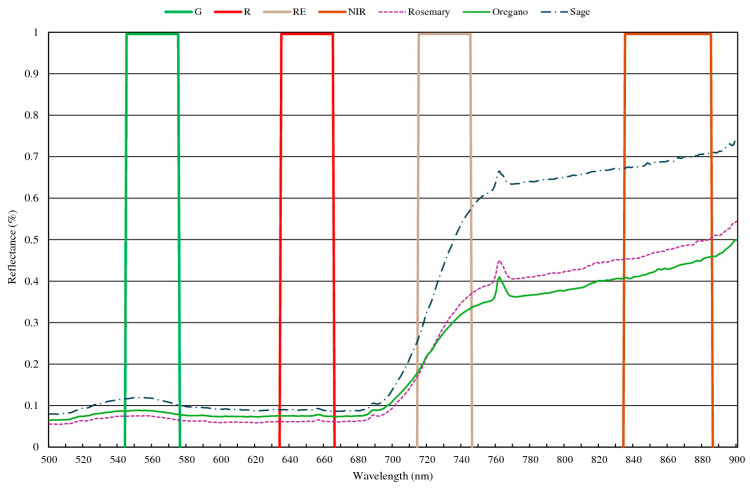
Reflectance of rosemary, oregano, and sage in the 500–900 nm spectrum region measured with the HH2 spectroradiometer and spectral sensitivity of the M3M camera (G—Green, R—Red, RE–Red Edge, and NIR—Near-Infrared).

The DJI Mavic 3 Multispectral (Dà-Jiāng Innovations Science and Technology Co., Ltd., Shenzhen, China) drone was used to capture high-resolution images across visible and near-infrared bands, including Green (G), Red (R), Red Edge (RE), and Near-Infrared (NIR) (Figure 5). The ASD FieldSpec HandHeld2 (HH2) spectroradiometer (Analytical Spectral Devices, Inc., Boulder, CO, USA) used in the tests has a sensitivity in the region 325–1075 nm, encompassing portions of the spectrum that are relevant for the characterization of the examined species. A solar irradiance sensor and a GNSS system with RTK correction (<2 cm accuracy) ensure precise data collection. Flight parameters were carefully planned to use DJI Pilot 2 software (Ver. 2.5) to avoid issues like shadows, with flights scheduled at noon for optimal lighting conditions in the period from March to October 2024.

Ground Control Points (GCPs) were placed and georeferenced using an RTK-enabled GNSS receiver. After image collection, data were processed in Agisoft Metashape Professional (Ver. 1.7.3) to create a multiband orthomosaic, which was then calibrated and orthorectified. The Ground Sampling Distance (GSD) of the orthomosaic obtained is 5 cm. Validation and accuracy enhancement of the orthomosaic were performed using eight Ground Control Points (GCPs) distributed across the field. The positions of the GCPs were determined using a GNSS receiver with RTK correction, ensuring a positional accuracy of ±2 cm.

Spectral canopy data were extracted and analyzed using QGIS software (Ver. 3.16), calculating NDVI values to monitor vegetation health and optimize harvest timing for MAPs [79,80,81]. To assess vegetation dynamics and health, NDVI values were calculated for three key periods: March, September, and October, to identify vegetation areas. Zonal statistics in QGIS were used to determine the surface area corresponding to different NDVI classes, providing valuable data for effective land management. Spectral data also facilitated the generation of false-color images, enhancing the visualization of vegetation and offering critical insights into the health and vigor of rosemary, oregano, and sage. The collected biomass was intended for the extraction of EOs process, so it was necessary to identify the time of highest vegetative vigor for MAPs. The NDVI index is sensitive toward crop biophysical properties, like nitrogen, chlorophyll, vigor, biomass, etc. In order to choose the optimal harvest time, the MAPs’ NDVI values were calculated in the period from 1st March to 30th October 2024 (Figure 1 and Figure 2). The collected leaves were tray-dried using a smart solar dryer system based on a Wireless Sensor Network (WSN) [80,81,82].

The Normalized Difference Vegetation Index (NDVI) is frequently favored over alternative vegetation indices, such as the Normalized Difference Red Edge (NDRE) and Soil-Adjusted Vegetation Index (SAVI), due to several significant advantages. Firstly, NDVI is one of the most widely employed vegetation indices for monitoring vegetation health and density, having undergone extensive validation that confirms its reliability across diverse applications, including crop monitoring and yield prediction [83,84,85,86]. Secondly, NDVI exhibits high sensitivity to variations in vegetation density and health, rendering it a robust indicator for evaluating plant growth conditions; this sensitivity allows it to effectively reflect the physiological and biophysical characteristics of vegetation, which is essential for precision agriculture [83,84,85]. Additionally, NDVI can be readily calculated from data obtained from various sources, including ground sensors, unmanned aerial vehicles (UAVs), airborne, and satellite sensors, which enhances its practicality for large-scale and long-term vegetation monitoring [86]. Furthermore, NDVI has demonstrated a strong correlation with critical indicators of crop yield and plant quality, particularly for medicinal and aromatic plants (MAPs) such as oregano, sage, and rosemary. Specifically, NDVI serves as a reliable tool for monitoring crop status and vigor, which are intrinsically linked to yield; studies indicate that NDVI can predict yields by evaluating overall vegetation health and density [86]. Moreover, NDVI is associated with various indices that reflect crop growth, including leaf area index (LAI), leaf nitrogen accumulation (LNA), and chlorophyll content, all of which are vital for assessing plant quality, including that of MAPs [85]. Lastly, the high temporal resolution of NDVI facilitates continuous monitoring of plant growth dynamics, which is crucial for understanding developmental stages and optimizing harvest times for enhanced yield quality [87,88].

#### 4.1.3. Extraction and Analyses of Essential Oils

The oregano EO was extracted from 500 g of dried leaves and flowers [79,80,89], using a 12 L conventional steam distiller for aromatic plants (Spring Extractor, Albrigi Luigi, Verona, Italy). After the distillation process, the EO was separated from its vegetation water and stored at 4 °C. Yield of EO was expressed in percentage (volume/weight).

The extraction of EO from rosemary was the same as that used for oregano, using 300 g of fresh plant material consisting mainly of flowering tops and leaves. After 3 h of distillation, a light yellow EO with camphor odor was obtained.

Chemical composition was determined by a GC/MS method using an Agilent 6890 Gas-Chromatography coupled with an Agilent 5973 Mass Spectrometer equipped with a DB1-MS silica capillary column (60 m × 0.25 mm, 0.5-micron film thickness). Injection of 1 microlite volume, with a split ratio 50:1, was applied. The oven temperature was held at 60 °C for 8 min, increased to 180 °C with a gradient of 4 °C/min, and then held for 2 min at 180 °C. The components were identified by helium as carrier gas (1 mL/min) and injector temperature and ion-source temperature were 250 °C and 280 °C, respectively. The identification of EOs compounds was performed by comparison with their relative retention time (RT) with those of original samples or by comparison with their relative retention index (RI) to the series of n-hydrocarbons. Linear RIs were determined by analyzing a standard solution of C7-C30 saturated alkanes and according to the equation proposed by Van den Dool and Kratz [90]. When a pure compound was not available, identification was based on the comparison of determined linear RIs with those reported by Adams [91] in the NIST Chemistry WebBook database [NIST Chemistry WebBook. Available online: https://webbook.nist.gov/chemistry/ (accessed on 1 February 2025)] and by a comparison of mass spectra with those reported in the NIST/EPA/NIH Mass Spectral Library (Version 2.4, 2020). The percent content of the compounds was determined from their peak areas in the GC Total Ion Current profile. Each oil sample was analyzed in duplicate.

For sage EO extraction and analysis, 200 g of leaves were manually chopped into small pieces and subjected to water distillation for three hours using a Clevenger apparatus. The extracted EO was dried with anhydrous sodium sulfate and stored in the dark at 4 °C until analysis. The EO was analyzed using a gas chromatograph-mass spectrometer (Shimadzu GC-MS QP2010 Ultra), following the method outlined by Zito et al. [92]. The GC-MS system was equipped with an AOC-20i self-injector (Shimadzu, Kyoto, Japan) and a ZB-5 column (5% phenyl-polysiloxane; 30 m length, 0.32 mm internal diameter, and 0.25 µm film thickness, Phnomenex). For each analysis, 1.3 µL of the sample was injected at 280 °C diluted in a 1:1 ratio, with a helium carrier gas flow rate set to 3 mL/min. The oven temperature was held at 60 °C for 1 min, then increased at a rate of 10 °C/min until reaching 300 °C, where it was maintained for 5 min. The MS interface was set to 300 °C, and the ion source operated at 200 °C. Mass spectra were recorded at 70 eV (EI mode) from 30 to 450 *m*/*z*. The GC-MS data were processed using the GC-MS Solution software, version 4.11 [35]. The yields of EOs extracted from the species under study were expressed as a percentage (volume/weight)

### 4.2. Aculops lycopersici Experimental Set-Up

#### 4.2.1. Solanum Nigrum Seedlings for *A. lycopersici* Breeding

Seeds of *S. nigrum* (black nightshade) were isolated from fruits gathered from field (38°6′25.03″ N, 13°21′0.19″ E), in autumn 2023, dried on filter paper for three days at room temperature and stored in a glass container in a refrigerator at 9 °C, 35% of RH and photoperiod of 0:24 (light/dark) for 6 months. In April 2024, seeds were sown in plastic pots (22 × 22 × 26 cm) using a substrate mixture of blonde peat and perlite and placed in a greenhouse.

The plants were grown in a controlled environment at a temperature of 25 °C and relative humidity ranging between 50% and 60%. The substrate consisted of a mixture of peat and potting soil, with a pH between 6.5 and 7. Irrigation was carried out manually once a day to maintain optimal moisture levels. Mites (*A. lycopersici*) were introduced three weeks after germination, when the first true leaves had fully expanded.

*Aculops lycopersici* was collected from *S. nigrum* plants in the garden of the Department SAAF in May 2024 and used for infesting *S. nigrum* potted plants placed inside entomological cages (150 × 150 mesh, 160 µm aperture), in a controlled condition (25 ± 1 °C, 70 ± 5% RH, 16:8 light/dark photoperiod).

#### 4.2.2. Adult Cohort for the Experiments

To obtain coetaneous for the experiments, 200 adults of *A. lycopersici* were transferred onto the adaxial surface of four *S. nigrum* leaves placed in Petri dishes (Ø150 mm, h 10 mm) on cotton wool saturated with distilled water. Adults were allowed to oviposit, and after a 24 h period, they were removed. The presence of juvenile stages was recorded daily, and the postembryonic development was monitored until attaining adulthood. Newly molted adults (max 48 h old) were used for the experiments. Since it was not possible to distinguish females from males under the stereomicroscope, a mixed population of both sexes was used in the trials.

#### 4.2.3. Experimental Units

The experimental unit (EU) consists of a leaf disc (Ø1.6 cm) placed on cotton wool moistened with distilled water in a Petri dish (Ø100 mm, h 10 mm). The adaxial surface of the leaf was used as experimental surface. The leaves used to obtain leaf discs for the experiments were taken from the apical portions of plants of similar age and position. The freshness of the discs was maintained by placing them on cotton wool saturated with distilled water. Freshly cut discs were used immediately after collection to ensure their freshness during the tests.

#### 4.2.4. Effects of Essential Oils on *A. lycopersici*

Five adults of *A. lycopersici* were transferred on each EU, using a specialized pen with micro clamping mandrel, into which a human eyelash was inserted at the tip. The flexibility of the eyelash and the presence of micro-sculptures on its surface enabled the delicate collection of the specimens without injuring them.

Essential oils were tested at five different concentrations: 320, 640, 1280, 2500, and 5000 µL L^−1^. Each EO was initially dissolved in pure acetone to ensure homogeneous mixing and afterwards distilled water was added in a 3:2 ratio (for water and acetone, respectively). For each concentration, 10 replications were carried out for a total of 50 *A. lycopersici* adults per test. The negative control replications were treated with only water and acetone at ratio 3:2.

Each replicate was treated with 8 mL of the solution using a Potter Precision Spray Tower (Burkard Manufacturing Co. Limited, Woodcock Hill Industrial Estate, Rickmansworth, Hertfordshire WD3 1PJ, England), set to a pressure of 62.05 kPa. The EUs were checked at 24 h intervals for 4 days after the treatment; the cotton wool was replenished daily with distilled water. Adult mortality was registered daily until the conclusion of the tests.

The range of concentrations tested was based on previous studies conducted on various phytophagous mite species, where these concentrations were found to be the most effective [25]. A preliminary test was conducted to evaluate the effects of water and acetone separately, but no mortality was observed in either treatment. Mites were considered dead after careful observation for approximately three minutes, during which they showed no response to mechanical stimulation (e.g., probing), lacked mobility, and often exhibited a faded or less vivid body coloration. The 10 replicates were conducted using essential oil solutions from the same batch, and leaf discs were taken from different plants of similar age and condition.

The categories proposed by Hardman et al. [36] for toxicity classes were applied on corrected mortality data [93]: (1) not toxic (mortality <25%), (2) slightly toxic (mortality between 26% and 50%), (3) moderately toxic (mortality between 51% and 75%), (4) very toxic (mortality >76%).

#### 4.2.5. Statistical Analysis

The Johnson and Kotz [94] angular transformation was applied to the mortality data before the general linear model analysis (GLM). The assumption of normality was verified on the basis of the analysis of residuals. The transformed mortality was included as the response variable, in respect of “Essential oils”, “concentrations”, and “time” (in days) as categorical covariates. In the presence of significant differences between treatments, the averages were separated by Tukey’s HSD test (*p* < 0.05). Mortality was corrected by Abbott’s [93] formula before the probit analysis. The number of dead mites was used as response in Event, the log-transformed concentrations as Stress (stimulus), and the essential oils as Factor, assuming the Weibull distribution and the maximum likelihood for estimation method. If the Pearson goodness-of-fit test was significant, each essential oil was analyzed singularly, eliminating the Factor from the model. The lethal concentrations corresponding to the mortality of 10% (LC_10_), 30% (LC_30_), 50% (LC_50_), and 90% (LC_90_) were determined by means of a probit model implemented in the Minitab software, considering a 95% confidence level. All analyses were performed using Minitab 19.0 software (Minitab Inc., State College, PA, USA).

## Figures and Tables

**Table 1:** Percentage of components characterizing the essential oils (EOs) of oregano, rosemary, and sage, with their relative weights (RW%).

Component/Chemical Class	Oregano EO (RW%)	Rosemary EO (RW%)	Sage EO (RW%)
**Monoterpene phenol**			
Carvacrol	**83.42**	-	-
Thymol	0.89	-	-
*Subtotal*	*84.31*	*-*	*-*
**Monoterpene hydrocarbons**			
ρ-Cymene	3.06	0.80	-
γ-Terpinene	2.93	2.10	-
β-Myrcene	1.01	1.21	2.19
α-Terpinene	0.99	0.45	-
Limonene	0.45	2.22	-
Terpinolene	0.41	0.53	-
β-Ocimene	0.42	-	-
β-Pinene	0.13	4.1	2.66
Sabinene	0.13	-	-
α-Pinene	-	**28.0**	2.66
Linalyl Acetate	-	1.12	-
Crisantenone	-	-	**12.87**
Camphene	-	**7.00**	**9.26**
*Subtotal*	*9.53*	*47.53*	*29.64*
**Oxygenated monoterpenes**			
Linalool	0.32	3.45	-
Camphor	0.27	6.20	**21.91**
Terpinen-4-ol	0.18	-	-
1,8-Cineole (Eucalyptol)	-	**11.00**	**27.67**
Borneol	-	**7.72**	2.59
β-Thujone	-	0.73	-
α-Thujone	-	-	5.32
Bornyl Acetate	-	-	1.45
4-Caranol	-	-	0.09
4-Terpineol	0.31	-	0.54
α-Terpineol	-	4.40	-
α-Thujene	0.23	-	-
α-Phellandrene	0.60	-	-
*Subtotal*	*1.91*	*33.37*	*59.57*
**Sesquiterpene hydrocarbons**			
β-Caryophyllene	1.07	6.64	1.46
α-Humulene	0.70	0.75	-
Germacrene	0.07	-	-
β-Bisabolene	0.63	-	-
Farnesene	0.40	-	-
α-Caryophyllene	-	-	0.89
Alloaromadendrene	-	-	0.24
α-Gurjunene	-	-	0.13
Carophyllene oxide		0.88	
*Subtotal*	*2.87*	*8.27*	*2.72*
**Oxygenated sesquiterpenes**			
Viridiflor	-	-	1.22
Palustrol	-	-	0.75
Ledol	-	-	0.53
Spathulenol	-	-	0.31
*Subtotal*	*-*	*-*	*2.81*
**Other compounds**			
Camphol	0.70	-	-
1-Octen-3-ol	0.49	-	-
3-Octanone	0.36	-	-
Carvacrol Methyl Ether	0.03	-	-
Maool	-	-	0.62
Diethyl Phthalate	-	-	0.53
Naphthalene	-	-	0.28
*Subtotal*	*1.58*	*-*	*1.43*

**Table 2:** Susceptibility of adult stages of *Aculops lycopersici* to different concentrations of *Origanum vulgare*, *Salvia rosmarinus*, and *Salvia officinalis*.

	Concentrations	Cumulative Mortality (%) (Mean ± SE)				Survival Time (Days)	Abbott’s Corrected Mortality	Toxicity Class
*Plant Extracts*	(µL L^−1^)	Day 1	Day 2	Day 3	Day 4	(Mean ± SE)	(%)	*
*O. vulgare*	5000	66.00 ± 7.92	74.00 ± 8.46	80.00 ± 7.30	90.00 ± 4.47 a	0.90 ± 0.203 a	89.13	**4**
	2500	14.00 ± 4.27	20.00 ± 2.98	34.00 ± 6.00	36.00 ± 7.77 bc	2.96 ± 0.216 bcd	30.43	**2**
	1280	0.00 ± 0.00	4.00 ± 2.67	12.00 ± 4.42	22.00 ± 5.54 efg	3.62 ± 0.114 ed	15.22	**1**
	640	2.00 ± 2.00	4.00 ± 2.67	12.00 ± 3.27	20.00 ± 5.16 ef	3.62 ± 0.124 ed	13.04	**1**
	320	2.00 ± 2.00	6.00 ± 3.06	10.00 ± 3.33	16.00 ± 4.00 efg	3.66 ± 0.127 ed	8.70	**1**
	Control	0.00 ± 0.00	0.00 ± 0.00	0.00 ± 0.00	8.00 ± 3.27 fg	3.92 ± 0.038 e	0.00	-
*S. rosmarinus*	5000	16.00 ± 6.53	36.00 ± 9.33	42.00 ± 9.17	46.00 ± 10.3 b	2.60 ± 0.234 b	46.00	**2**
	2500	12.00 ± 4.42	24.00 ± 4.99	34.00 ± 4.27	40.00 ± 5.16 bc	2.90 ± 0.214 bcd	40.00	**2**
	1280	2.00 ± 2.00	12.00 ± 4.42	20.00 ± 5.16	26.00 ± 3.06 de	3.40 ± 0.159 cde	26.00	**2**
	640	2.00 ± 2.00	16.00 ± 4.99	16.00 ± 4.99	22.00 ± 6.96 e	3.44 ± 0.165 cde	22.00	**1**
	320	4.00 ± 2.67	6.00 ± 3.06	6.00 ± 3.06	12.00 ± 4.42 efg	3.72 ± 0.128 cd	12.00	**1**
	Control	0.00 ± 0.00	0.00 ± 0.00	0.00 ± 0.00	0.00±0.00 g	4.00 ± 0.00 e	0.00	-
*S. officinalis*	5000	12.00 ± 5.33	32.00 ± 9.52	42.00 ± 9.17	42.00 ± 9.17 bc	2.72 ± 0.225 bc	39.58	**2**
	2500	12.00 ± 6.80	32.00 ± 6.80	40.00 ± 7.30	54.00 ± 8.46 b	2.62 ± 0.216 bc	52.08	**3**
	1280	10.00 ± 4.47	28.00 ± 6.11	32.00 ± 8.00	34.00 ± 6.70 bcd	2.96 ± 0.218 bcd	31.25	**2**
	640	0.00 ± 0.00	14.00 ± 4.27	22.00 ± 5.54	22.00 ± 5.54 cde	3.42 ± 0.159 cde	18.75	**1**
	320	4.00 ± 2.67	16.00 ± 4.00	18.00 ± 4.67	28.00 ± 3.27 bcde	3.34 ± 0.173 bcde	25.00	**1**
	Control	0.00 ± 0.00	0.00 ± 0.00	0.00 ± 0.00	4.00 ± 2.67 e	3.96 ± 0.028 e	0.00	-

Different letters indicate significant differences among extracts and concentrations for survival time and corrected mortality. Tukey’s multiple comparison tests (*p* < 0.05) were applied after GLM analysis. * Toxicity classes (green: non-toxic, yellow: slightly toxic, orange: moderately toxic, red: highly toxic) were defined on corrected mortality after Hardman et al. [36].

**Table 3:** Lethal concentrations of the essential oils of *Origanum vulgare*, *Salvia rosmarinus*, and *Salvia officinalis* against adult stages of *Aculops lycopersici*.

*Essential Oils*	LC_10_ mL L^−1^ (95% CI)	LC_30_ mL L^−1^ (95% CI)	LC_50_ mL L^−1^ (95% CI)	LC_90_ mL L^−1^ (95% CI)	LC_95_ mL L^−1^ (95% CI)	Intercept ± SE	Slope ± SE	Goodness of fit χ^2^ (d.f.)
*Origanum vulgare*	0.369 (0.209–0.530)	1.068 (0.800–1.362)	2.229 (1.740–3.051)	13.452 (8.015–31.805)	22.392 (12.085–63.218)	−5.49 ± 0.71	1.64 ± 0.22	21.62 (3) *p* = 0.000
*Salvia rosmarinus*	0.207 (0.034–0.428)	1.489 (0.908–2.452)	5.835 (3.281–22.289)	164.369 (35.154–10573.530)	423.448 (67.507–61877.107)	−3.32 ± 0.67	0.88 ± 0.21	0.53 (3) *p* = 0.911
*Salvia officinalis*	0.027 (0.000002–0.146)	0.655 (0.080–1.292)	6.014 (2.630–261.607)	1358.595 (74.342–20,379,803,862.871)	6315.387 (184.714–3651,741,272,548.380)	−2.05 ± 0.61	0.54 ± 0.19	5.73 (3) *p* = 0.125

## Data Availability

Data is contained within the article.
